# Stereocomplex micelle from nonlinear enantiomeric copolymers efficiently transports antineoplastic drug

**DOI:** 10.1186/s11671-015-0907-2

**Published:** 2015-05-06

**Authors:** Jixue Wang, Kexin Shen, Weiguo Xu, Jianxun Ding, Xiaoqing Wang, Tongjun Liu, Chunxi Wang, Xuesi Chen

**Affiliations:** Department of Urology, the First Hospital of Jilin University, 71 Xinmin Street, Changchun, 130021 People’s Republic of China; Department of Urology, the First Hospital of Jilin University, 71 Xinmin Street, Changchun, 130021 People’s Republic of China; Key Laboratory of Polymer Ecomaterials, Changchun Institute of Applied Chemistry, Chinese Academy of Sciences, 5625 Renmin Street, Changchun, 130022 Peolple’s Republic of China

**Keywords:** Antineoplastic drug, Controlled delivery, Enantiomeric copolymers, Malignancy therapeutic, Stereocomplex micelle

## Abstract

Nanoscale polymeric micelles have attracted more and more attention as a promising nanocarrier for controlled delivery of antineoplastic drugs. Herein, the doxorubicin (DOX)-loaded poly(D-lactide)-based micelle (PDM/DOX), poly(L-lactide)-based micelle (PLM/DOX), and stereocomplex micelle (SCM/DOX) from the equimolar mixture of the enantiomeric four-armed poly(ethylene glycol)-polylactide (PEG-PLA) copolymers were successfully fabricated. In phosphate-buffered saline (PBS) at pH 7.4, SCM/DOX exhibited the smallest hydrodynamic diameter (*D*_h_) of 90 ± 4.2 nm and the slowest DOX release compared with PDM/DOX and PLM/DOX. Moreover, PDM/DOX, PLM/DOX, and SCM/DOX exhibited almost stable *D*_h_s of around 115, 105, and 90 nm at above normal physiological condition, respectively, which endowed them with great potential in controlled drug delivery. The intracellular DOX fluorescence intensity after the incubation with the laden micelles was different degrees weaker than that incubated with free DOX · HCl within 12 h, probably due to the slow DOX release from micelles. As the incubation time reached to 24 h, all the cells incubated with the laden micelles, especially SCM/DOX, demonstrated a stronger intracellular DOX fluorescence intensity than free DOX · HCl-cultured ones. More importantly, all the DOX-loaded micelles, especially SCM/DOX, exhibited potent antineoplastic efficacy *in vitro*, excellent serum albumin-tolerance stability, and satisfactory hemocompatibility. These encouraging data indicated that the loading micelles from nonlinear enantiomeric copolymers, especially SCM/DOX, might be promising in clinical systemic chemotherapy through intravenous injection.

## Background

In recent years, a variety of nanoscale polymeric drug delivery systems (DDSs) have made great strides in the design and fabrication, such as micelles [1,2], vesicles [3,4], nanogels [5,6], and so on. Among them, micelles as a promising type of DDSs have attracted widely attention for their satisfactory solubilization, targetability, P-glycoprotein inhibition, and efficient drug endocytosis [7]. Micelles are constructed through the spontaneous self-assembly of amphiphilic copolymers in an aqueous condition [8,9]. In addition, micelles exhibit a core-shell architecture, that is, they are composed of an inner core and an outer shell [10]. The outer shell is related to the pharmacokinetic behavior *in vivo*, and the inner core affects stability, drug loading efficiency, and release behavior [7]. The particular structural feature contributes to the satisfactory characteristics mentioned above.

Despite micelles have achieved great progress in the past few decades, the stability of them is still a major impediment for their extensive application in the field of controlled drug delivery [11]. Consequently, various weak interplays, such as hydrophobic, electrostatic, host-guest, and stereocomplex interactions, have been explored to enhance the stability of micelle and overcome other obstacles simultaneously [10]. Among them, the stereocomplex interaction-assisted polymeric micelles have gained increasing attention for their improved thermal and mechanical resistance, elevated stability, and reduced degradation rate [12,13]. Typically, the enantiomeric poly(L-lactide) (PLLA) and poly(D-lactide) (PDLA) copolymers have been the most widely explored polymeric matrices for stereocomplexation since Ikada *et al.* first reported them in 1987 [14,15]. Chen and coworkers prepared the stereocomplex micelles (SCMs) of the enantiomeric poly(ethylene glycol)-polylactide (PEG-PLA) block copolymers with different lengths of PLA chains for rifampin delivery, which showed lower critical micelle concentrations (CMCs), smaller micelle sizes, and higher loading capacities and encapsulation efficiencies compared with the micelles with a single component [16]. Moreover, the release performance of the drug-loaded SCM was related to the molecular weight and composition of block copolymer, and the morphology of micelle. Recently, the same group prepared the SCMs based on amphiphilic dextran-*block*-polylactide (Dex-*b*-PLA) copolymers, which could deliver DOX with high stability and sustained release profiles *in vitro* [17]. Moreover, in our previous work, a DOX-loaded SCM based on four-armed PEG-*b*-PLA was fabricated and exhibited a better antineoplastic effect than the laden micelles with a single matrix [18]. However, the previous platforms exhibit some limitations, such as complex synthesis process and large diameter.

In the current work, in order to further verify the impact of stereocomplex on the performance of micelle and optimize the performances of SCM, the DOX-loaded SCM (i.e., SCM/DOX) based on equimolar four-armed PEG-PDLA/PEG-PLLA exhibiting different molecular weights and compositions with previous systems was prepared by nanoprecipitation. The DOX-loaded micelles derived from individual four-armed PEG-PDLA and PEG-PLLA were referred as PDM/DOX and PLM/DOX, respectively. The laden micelles exhibited stable morphologies in normal physiological condition and could be efficiently internalized by RenCa cells (a mouse renal carcinoma cell line). Moreover, all these formulations, especially SCM/DOX, showed excellent anti-proliferative efficiency toward RenCa cells, which were even better than free DOX · HCl. In addition, all the loading micelles maintained excellent serum albumin-tolerance stability and showed lower hemolytic rates compared with free DOX · HCl, which endowed them with great promise of applying *in vivo*.

## Methods

### Materials

Four-armed PEG (number-average molecular weight (*M*_n_) = 10,000 Da) was obtained from Shanghai Seebio Biotech, Inc. (Shanghai, People’s Republic of China) and used as received. DLA and LLA were provided by Changchun SinoBiomaterials Co., Ltd. (Changchun, People’s Republic of China) and recrystallized from ethyl acetate under argon atmosphere before use. Four-armed PEG-PDLA and PEG-PLLA were synthesized as our previously reported proposal [18]. The degree of polymerization (DP) of PLA in each arm was calculated to be 16 based on the data of proton nuclear magnetic resonance (^1^H NMR). The *M*_n_ of copolymer was estimated to be 14,600 g mol^−1^. Doxorubicin hydrochloride (DOX · HCl) was purchased from Beijing HuaFeng United Technology Co., Ltd. (Beijing, People’s Republic of China). 4′,6-Diamidino-2-phenylindole (DAPI), Alexa Fluor 488 phalloidin (Alexa 488), and 3-(4,5-dimethylthiazol-2-y1)-2,5-diphenyltetrazolium bromide (MTT) were purchased from Sigma-Aldrich (Shanghai, People’s Republic of China). RenCa cell line was obtained from the American Type Culture Collection. Clear 6-well and 96-well tissue culture polystyrene (TCP) plates were purchased from Corning Costar Co. (Cambridge, MA, USA). The deionized water used in this study was prepared through a Milli-Q water purification equipment (Millipore Co., MA, USA).

### Encapsulation of DOX

The encapsulation of DOX into these micelles was performed through a nanoprecipitation method, which was described in previous studies [19,20]. Typically, 100.0 mg of four-armed PEG-PDLA copolymer was dissolved in 10.0 mL of *N*,*N*-dimethylformamide (DMF) (i.e., 10.0 mg mL^−1^), and then DOX · HCl (21.3 mg) dissolved in 6.0 mL of Milli-Q water was added dropwisely. After that, 2.0 mL of phosphate-buffered saline (PBS) was added into the mixed solution to neutralize the hydrochloric acid in DOX · HCl. The mixture was stirred at room temperature for 12 h and subsequently dialyzed using a dialysis bag (molecular weight cut-off (MWCO) = 3,500 Da) against deionized water for 12 h. After freeze-drying, PDM/DOX was obtained. PLM/DOX and SCM/DOX were fabricated in the same way.

For determining the drug loading content (DLC) and drug loading efficiency (DLE), the DOX-loaded micelles were dissolved in DMF and stirred for 12 h at room temperature in the dark. And then, the amount of DOX in micelles were analyzed with fluorescence measurements using a standard curve method on a Photon Technology International (PTI) Fluorescence Master System with software Felix 4.1.0 (λ_ex_ = 480 nm; PTI, Inc., Lawrenceville, NJ, USA). The DLC and DLE of the DOX-loaded micelles were calculated according to the following Formulas (1) and (2), respectively.

1 $$ \mathrm{D}\mathrm{L}\mathrm{C}\;\left(\mathrm{wt}.\%\right)=\frac{\mathrm{Weight}\kern0.24em \mathrm{of}\kern0.24em \mathrm{drug}\kern0.24em \mathrm{in}\kern0.24em \mathrm{micelle}}{\mathrm{Weight}\kern0.24em \mathrm{of}\kern0.24em \mathrm{drug}\hbox{-} \mathrm{loaded}\;\mathrm{micelle}}\times 100 $$

2 $$ \mathrm{D}\mathrm{L}\mathrm{E}\;\left(\mathrm{wt}.\%\right)=\frac{\mathrm{Weight}\kern0.24em \mathrm{of}\kern0.24em \mathrm{drug}\kern0.24em \mathrm{in}\kern0.24em \mathrm{micelle}}{\mathrm{Total}\kern0.24em \mathrm{weight}\kern0.24em \mathrm{of}\kern0.24em \mathrm{feeding}\;\mathrm{drug}}\times 100 $$

### Measurements

The morphologies and sizes of the DOX-loaded micelles were characterized by transmission electron microscopy (TEM) and dynamic light scattering (DLS). For TEM observation, 10.0 μL of the loading micelle solution (0.1 mg mL^−1^) was placed on a copper grid and air-dried at room temperature. The tests were carried out on a JEOL JEW-1011 instrument (JEOL, Peabody, MA, USA) operating at an accelerating voltage of 100 kV. For DLS assays, the DOX-loaded micelles were dissolved in PBS at pH 7.4, and the *D*_h_s of the laden micelles with different incubation times were detected at 25°C on a WyattQELS apparatus. The intensity results were used, and the average histograms were shown. The PDI of *D*_h_ was referred as the ratio of standard deviation and the mean of *D*_h_.

### *In vitro* DOX release

The release profiles of the DOX-loaded micelles were studied using a dialysis bag (MWCO = 3,500 Da) at pH 7.4. In brief, 1.0 mg of PDM/DOX, PLM/DOX, or SCM/DOX was dissolved in 10.0 mL of PBS and then transferred into the dialysis bag (MWCO = 3,500 Da). The dialysis bag was then put into 100.0 mL of PBS at 37°C with continuous vibrations of 70 rpm. At desired time intervals, 2.0 mL of release medium was taken out, and an equal volume of PBS was replenished. The release of DOX was determined by fluorescence spectroscopy.

### Intracellular DOX release analyses

The cellular uptakes of the DOX-loaded micelles and free DOX · HCl were qualitatively detected by confocal laser scanning microscopy (CLSM) and quantitatively estimated by flow cytometry (FCM) toward RenCa cells.

#### CLSM

The RenCa cells were seeded on glass coverslips at a density of 2.0 × 10^5^ cells per well in 2.0 mL of complete high glucose Dulbecco’s modified Eagle’s medium (HG-DMEM) in 6-well plates for 24 h. PDM/DOX, PLM/DOX, SCM/DOX, or free DOX · HCl with a DOX · HCl dosage of 10.0 μg mL^−1^ was added to each well. After coincubation for 2 h, the medium was removed, and the cells on glass coverslips were washed with PBS five times and immobilized by 4% (*W*/*V*) PBS-buffered paraformaldehyde for 20 min at room temperature. And then, the cells were washed with PBS five times and reacted with 0.1% (*V*/*V*) Triton X-100 in PBS for 12 min at room temperature. The nuclei were then stained with DAPI for 3 min at 37°C, after which the cells were washed with PBS five times. At last, the filamentous actin was stained with Alexa 488 for 30 min at 37°C and washed with PBS five times. The fluorescence was observed with a LSM 780 CLSM (Carl Zeiss, Jena, Germany). Additionally, to further monitor the intracellular DOX release of these micelles with the time, PDM/DOX, PLM/DOX, SCM/DOX, or free DOX · HCl with a DOX · HCl dosage of 1.0 μg mL^−1^ was coincubated with the cells. After coculture for 2, 6, 12, or 24 h, the cells were detected as mentioned above.

#### FCM

RenCa cells were seeded in 6-well plates at a density of 2.0 × 10^5^ cells per well and cultured with 2.0 mL of complete HG-DMEM for 24 h. And then, PDM/DOX, PLM/DOX, SCM/DOX, or free DOX · HCl with a DOX · HCl dosage of 10.0 μg mL^−1^ was added to each well. The cells without any treatment were set as control. After a 2 h coculture, the medium was removed, and the cells were washed five times with PBS and then digested by trypsin. Subsequently, 1.0 mL of PBS was added and collected in centrifuge tubes for centrifugation at 3,500 rpm for 5 min. After removing the supernatants, the bottom cells were resuspended in 0.3 mL of PBS and examined by a flow cytometer (λ_ex_ = 488 nm; Beckman, CA, USA).

### Cytotoxicity assays

The cytotoxicities of blank micelles were tested by a MTT assay on RenCa cells with a final concentration from 1.6 to 100.0 μg mL^−1^. In brief, the cells were seeded on a 96-well plate at a density of 8.0 × 10^3^ cells in 180.0 μL of HG-DMEM and incubated at 37°C for 24 h. And then, 20.0 μL of micelle solutions at different concentrations were added to each well and cultured for another 48 h. Subsequently, 20.0 μL of MTT at a concentration of 5.0 mg mL^−1^ was added to each well. After incubation for another 4 h, the medium was carefully removed, and 150.0 μL of dimethyl sulfoxide (DMSO) was added. After vibration for 5 min, the absorbance of medium was determined at 490 nm using a Bio-Rad 680 microplate reader. In addition, the *in vitro* antineoplastic activities of the DOX-loaded micelles and free DOX · HCl with a DOX · HCl dosage of 0.16 to 10.0 μg mL^−1^ were also assessed on RenCa cells following the above protocol. The cell viability was calculated as Formula (3).

3 $$ \mathrm{Cell}\;\mathrm{viability}\ \left(\%\right)=\frac{A_{\mathrm{sample}}}{A_{\mathrm{control}}}\times 100 $$

In Formula (3), *A*_sample_ and *A*_control_ represented the absorbances of sample and control wells, respectively.

### Serum albumin-tolerance stability assays

The stability of the laden micelles in the PBS-buffered bovine serum albumin (BSA) solution (30.0 mg mL^−1^) at pH 7.4, 25°C was tested through DLS at different time points.

### Hemolytic activity tests

The hemolytic activities of PDM/DOX, PLM/DOX, SCM/DOX, and free DOX · HCl were tested by a spectrophotometry technique. Typically, the fresh rabbit blood obtained from the Experimental Animal Center of Jilin University was stabilized with dipotassium ethylene diamine tetraacetate (EDTAP) in normal saline (NS). After being centrifuged at 1,500 rpm for 10 min, the obtained red blood cells (RBCs) were carefully washed and diluted with NS. And then, the various concentrations of PDM/DOX, PLM/DOX, SCM/DOX, and free DOX · HCl were added into the suspension of RBCs at 37°C for 2 h. NS was used as negative control, and Triton X-100 (i.e., a lysing agent of RBCs) was used as positive control. After centrifugation at 3,000 rpm for 10 min, 180.0 μL of the supernatant from each sample was collected into a 96-well plate. Then the free hemoglobin in supernatant was tested at 570 nm by a Bio-Rad 680 microplate reader (Hercules, CA, USA). The hemolytic ratio of RBCs was calculated as Formula (4).

4 $$ \mathrm{Hemolytic}\;\mathrm{ratio}\ \left(\%\right)=\frac{A_{\mathrm{sample}}-{A}_{\mathrm{negative}\ \mathrm{control}}}{A_{\mathrm{positve}\ \mathrm{control}}-{A}_{\mathrm{negative}\ \mathrm{control}}}\times 100 $$

In Formula (4), *A*_sample_, *A*_negative control_, and *A*_positive control_ represented the absorbances of sample, and negative and positive controls, respectively.

### Statistical analyses

All tests were carried out independently at least three times, and the data were expressed as mean ± standard deviation (SD). The data were analyzed for statistical significance using SPSS (Version 13.0, USA). ^&^*p* < 0.05 was considered statistically significant, and ^#^*p* < 0.01 and *p < 0.001 were considered highly significant.

## Results and discussion

### Preparations and characterizations of DOX-loaded micelles

The branched and multi-armed copolymers have gained more and more attention for their more excellent rheological, mechanical, and biomedical properties than the linear ones [21-23]. In this study, two enantiomeric four-armed copolymers of PEG-PDLA and PEG-PLLA were explored as the matrices of nanocarriers for the controlled delivery of antineoplastic drug into RenCa cells. The four-armed PEG-PDLA and PEG-PLLA, and an equimolar mixture of the above two copolymers were prone to self-assemble into micelles in aqueous solution because of the amphiphilic nature, which were noted as PDM, PLM, and SCM, respectively. The CMCs of PDM, PLM, and SCM were detected to be 4.5 × 10^−3^, 3.6 × 10^−3^, and 1.7 × 10^−3^ mg mL^−1^, respectively. Compared with PDM and PLM, SCM showed the lowest CMC, which should be attributed to the formation of tightly packed hydrophobic core enhanced by the stereocomplex interaction.

**Figure 1:**
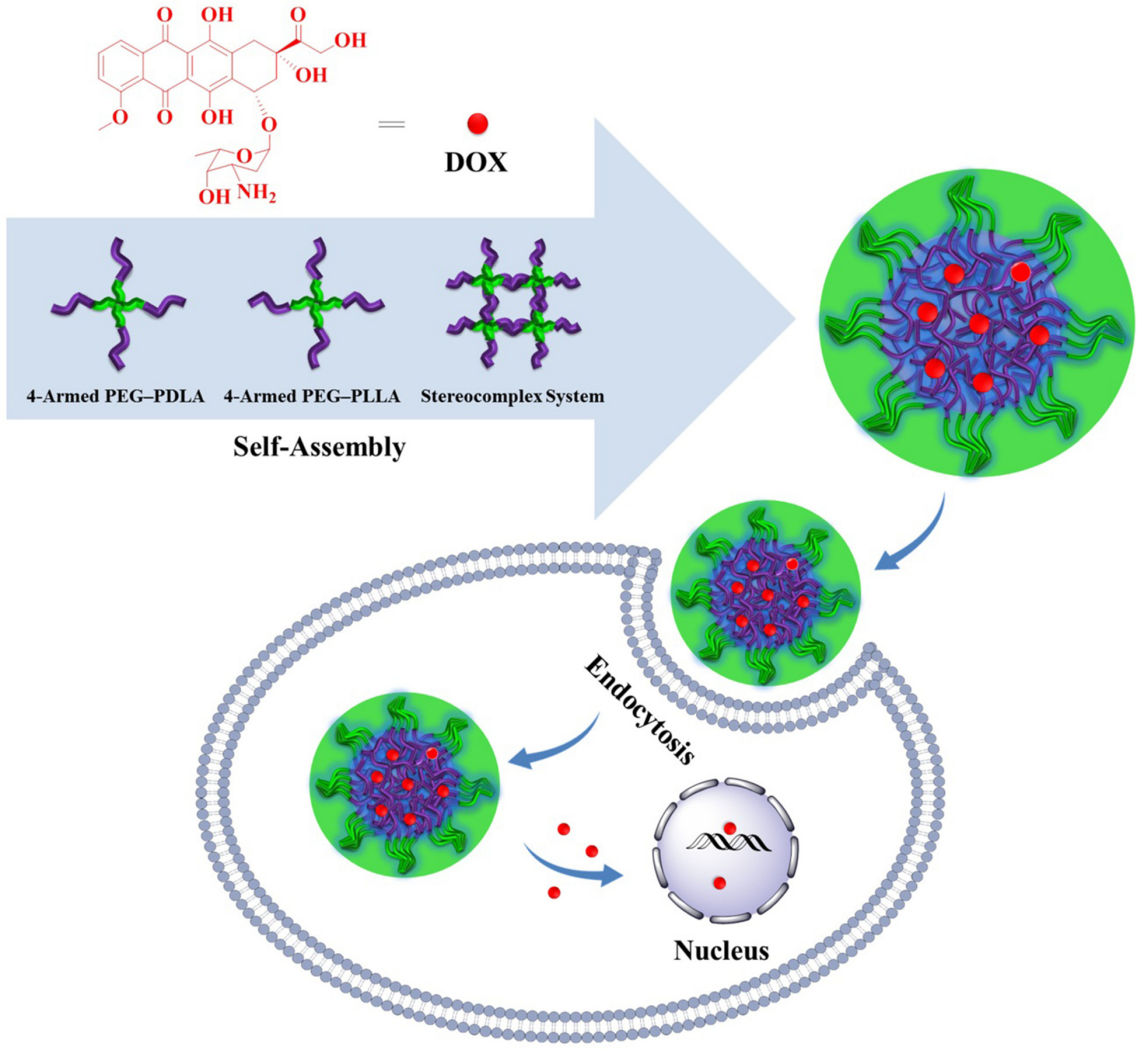
Schematic illustration for construction of PDM/DOX, PLM/DOX, and SCM/DOX, and cellular uptakes and intracellular DOX release in RenCa cells.

**Figure 2:**
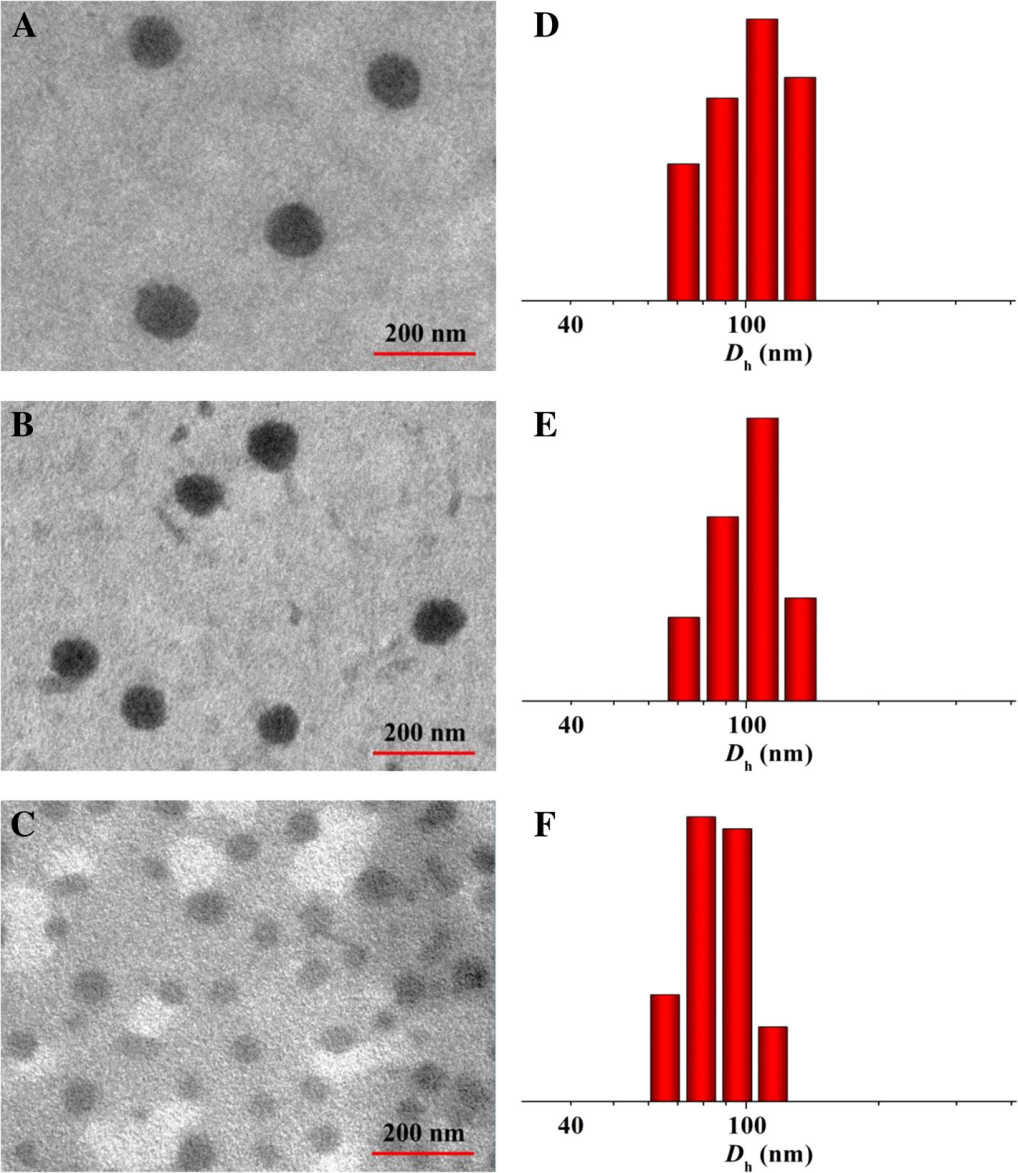
Typical TEM micrographs **(A, B, and C)** and *D*_h_s **(D, E, and F)** of PDM/DOX **(A and D)**, PLM/DOX **(B and E)**, and SCM/DOX **(C and F)**.

**Figure 3:**
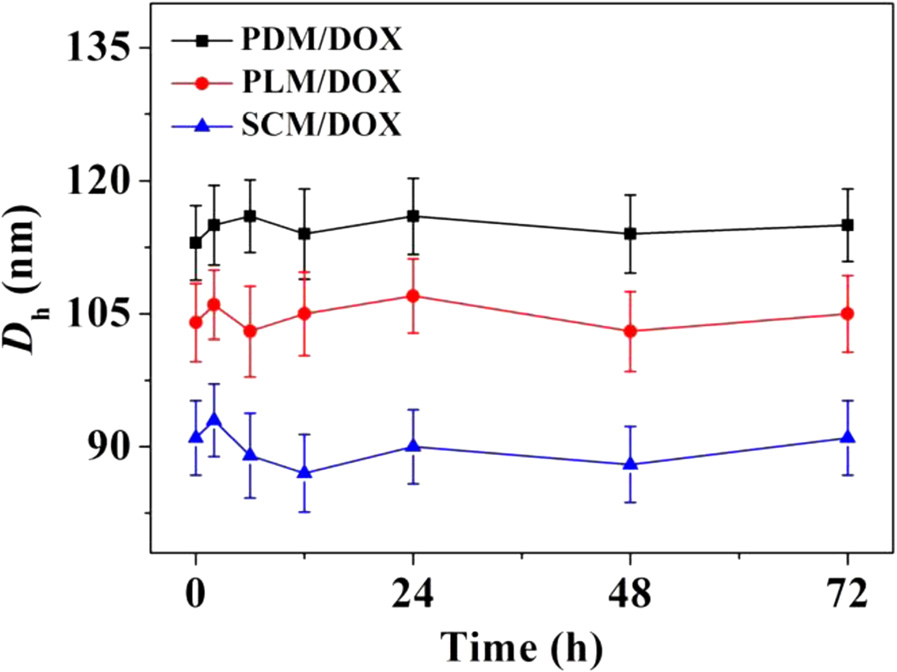
*D*_h_ changes of PDM/DOX, PLM/DOX, and SCM/DOX versus time in PBS at pH 7.4, 25°C. Each datum was represented as mean ± SD (*n* = 3).

A model antineoplastic agent, i.e., DOX, was physically encapsulated into PDM, PLM, or SCM through nanoprecipitation, and the DOX-loaded micelles were referred as PDM/DOX, PLM/DOX, and SCM/DOX, respectively. The process was illustrated in Figure 1. The DLCs of PDM/DOX, PLM/DOX, and SCM/DOX were calculated to be 8.5, 8.9, and 10.1 wt.%, and the DLEs of them were 43.6, 45.9, and 52.7 wt.%, respectively. It indicated that the stable stereocomplex crystallization of enantiomeric PLA in micellar core induced higher DLC and DLE of SCM/DOX [16]. The morphologies and sizes of the DOX-loaded micelles were characterized by TEM and DLS. As shown in Figure 2A, 2B, and 2C, the TEM micrographs showed that PDM/DOX, PLM/DOX, and SCM/DOX took clear spherical morphologies with the respective average sizes of approximately 100, 90, and 75 nm, respectively. In contrast, the DLS measurements showed that the mean diameters of laden micelles were 115 ± 4.6, 105 ± 4.4, and 90 ± 4.2 nm, respectively (Figure 2D, 2E, and 2F). The SCM/DOX exhibited the smallest size. This might be related to the formation of stereocomplex, which strongly improved the stability of copolymer [17,24]. Moreover, the polydispersity indices (PDIs) of *D*_h_s were calculated to be 0.15, 0.14, and 0.12, respectively, which indicated the narrow size distribution of these loading micelles. Bigger values from DLS detections compared with those from TEM measurements should be attributed to the hydration state of micelles [25]. The sizes of these laden micelles were appropriate for them to passively target in tumor tissues through the enhanced permeability and retention (EPR) effect [26]. Moreover, as shown in Figure 3, all these laden micelles demonstrated nearly constant diameters during the incubation in PBS at pH 7.4 for 72 h, which indicated the excellent stability of the test laden micelles.

### Drug release profiles from laden micelles

**Figure 4:**
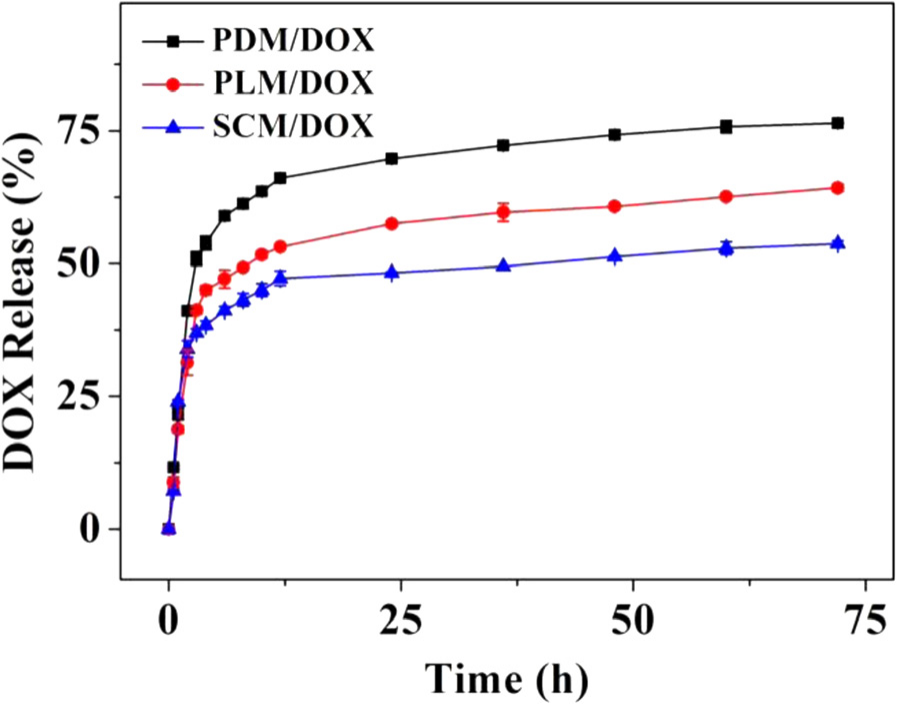
Release behaviors of PDM/DOX, PLM/DOX, and SCM/DOX in PBS at pH 7.4, 37°C. Each datum was represented as mean ± SD (*n* = 3).

The release of DOX from laden micelles was investigated in PBS at pH 7.4, 37°C (Figure 4). It could be observed that the release behaviors of PDM/DOX and PLM/DOX showed three phases: i) a fast release stage, in which about 55 and 45% of loaded DOX were released during the first 5 h, respectively; ii) a continuously slow release phase, in which 65 and 55% of loaded DOX were released before 25 h, respectively; iii) a platform period, in which only a little loaded DOX was released until 72 h. The initial fast release was considered to be relevant to the absorption of DOX by the shallow parts of micelles. The slow release behavior might be attributed to the diffusion of DOX through the laden micelles and the degradation of PLA block [27]. Compared with PDM/DOX and PLM/DOX, SCM/DOX exhibited weaker initial burst release and slower sustained release rate in 72 h, which might be relevant to the enhanced stability of SCM [24]. Therefore, SCM represented as a great promising approach to efficiently load and controllably release antineoplastic drugs.

**Figure 5:**
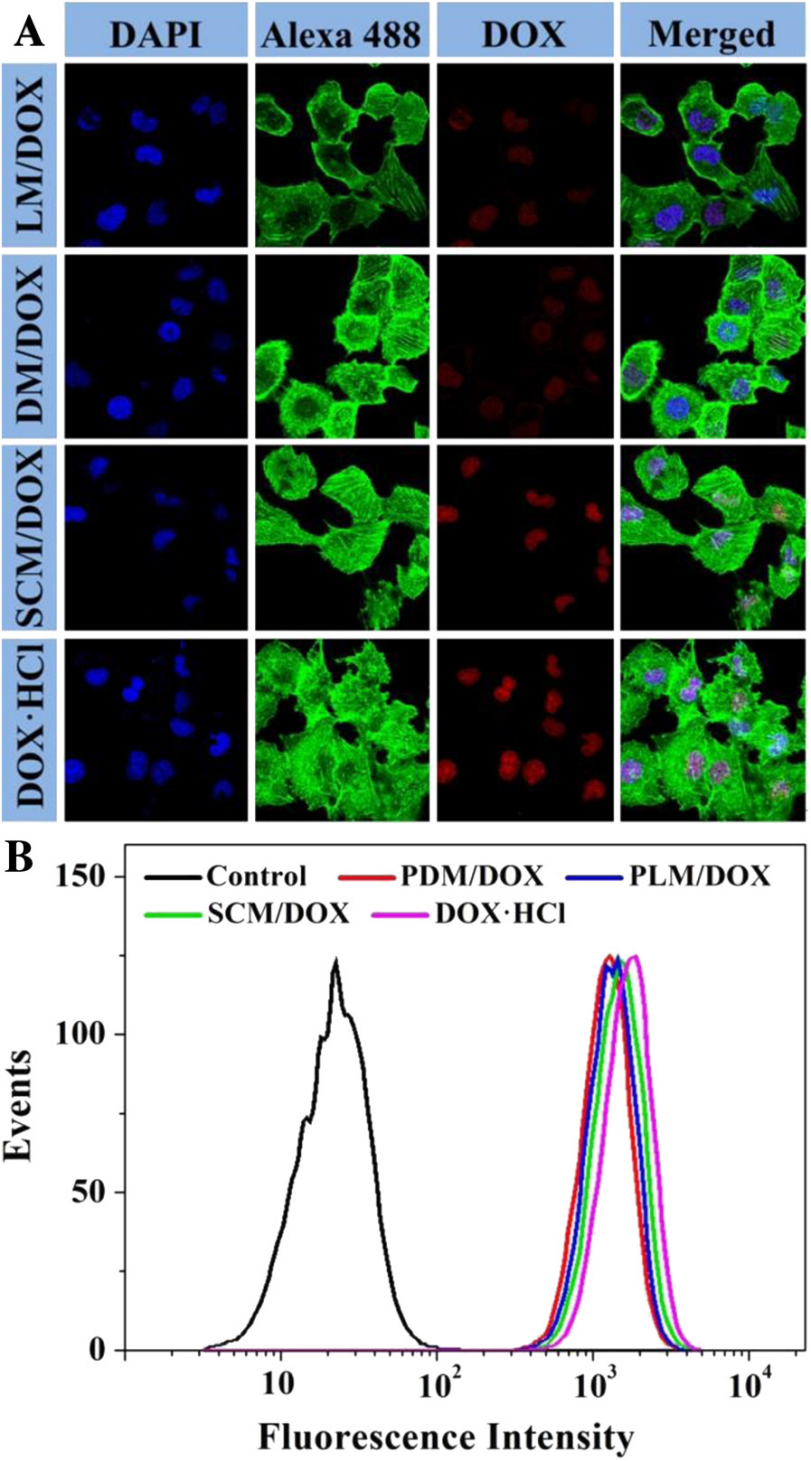
Typical CLSM microimages **(A)** and FCM determinations **(B)** of RenCa cells incubated with PDM/DOX, PLM/DOX, SCM/DOX, or free DOX · HCl for 2 h.

The cellular uptakes and intracellular release behaviors of the DOX-loaded micelles toward RenCa cells were evaluated using CLSM and FCM to validate whether these laden micelles were effective to deliver DOX into cells. From the CLSM microimages in Figure 5A, a stronger intracellular DOX fluorescence intensity was observed after incubation with SCM/DOX for 2 h than those with the DOX-loaded micelles with single component. This result was possibly relevant to slower extracellular DOX release and more efficient intracellular DOX release. In contrast, the intracellular DOX fluorescence intensity of the free DOX · HCl group was a little stronger than that of the SCM/DOX one after coincubation for 2 h. It was because that the cellular uptake of free DOX · HCl by diffusion was faster than the endocytosis of DOX-incorporated micelles in a relatively short detection time [28]. To further prove our statement, the FCM histograms of the loading micelles and free DOX · HCl were analyzed. As shown in Figure 5B, the signal intensity of SCM/DOX was stronger than those of PDM/DOX and PLM/DOX, and there was little difference between those of the PDM/DOX and PLM/DOX groups. The free DOX · HCl group exhibited the highest fluorescence intensity after coculture for 2 h that was agreed very well with the results of CLSM. The data of CLSM and FCM proved the effective internalization of the laden micelles by RenCa cells.

**Figure 6:**
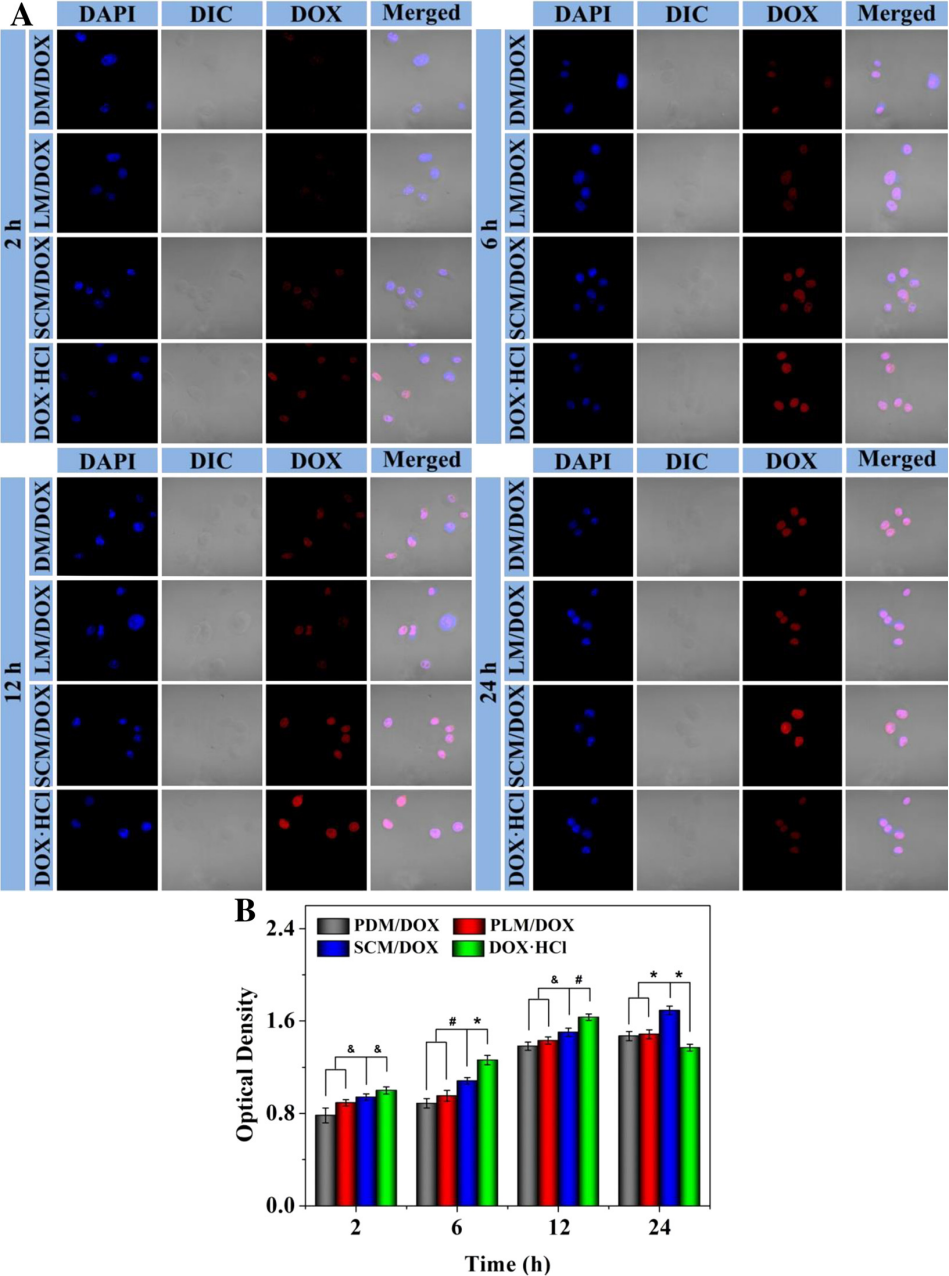
Intracellular DOX internalization of PDM/DOX, PLM/DOX, SCM/DOX, and free DOX · HCl groups after coculture for different periods of time **(A)**, and statistical analysis of optical density of DOX fluorescence intensity **(B)**. The data were shown as mean ± SD (*n* = 4; ^&^ *p* < 0.05, ^#^ *p* < 0.01, and *p < 0.001).

In addition, a relatively low dosage of DOX · HCl, i.e., 1.0 μg mL^−1^, was used for testing the intracellular DOX release within 24 h, which aimed to avoid the death of cells with a longer time of incubation. As shown in Figure 6A, the free DOX · HCl group exhibited the highest intracellular DOX fluorescence intensity compared with the laden micelle ones after incubation for 2 h (*p* < 0.05). Until 12 h, the intracellular DOX fluorescence intensity of the free DOX · HCl group was stronger than those of the loading micelle groups (*p* < 0.05), although the laden micelle groups showed a gradually increased intensity with the extension of time. More interestingly, the results were reversed after 24-h culture, that is, the DOX-loaded micelle groups, especially, the SCM/DOX one, showed stronger intracellular DOX fluorescence intensity than the free DOX · HCl group (*p* < 0.001). The SCM/DOX group exhibited a higher signal than those of the laden micelles with single component for all the test time points (*p* < 0.05). Moreover, the quantification of DOX fluorescence intensity was shown in Figure 6B. The relative optical density of DOX fluorescence intensity was analyzed with ImageJ software (National Institutes of Health, Bethesda, Maryland, USA). The intracellular DOX fluorescence intensity of the free DOX · HCl group after coincubation for 2 h was set as “1”. These results further demonstrated that these laden micelles, especially SCM/DOX, could achieve efficiently intracellular DOX release with a longer intracellular responding time.

### *In vitro* antineoplastic activities toward RenCa cells

**Figure 7:**
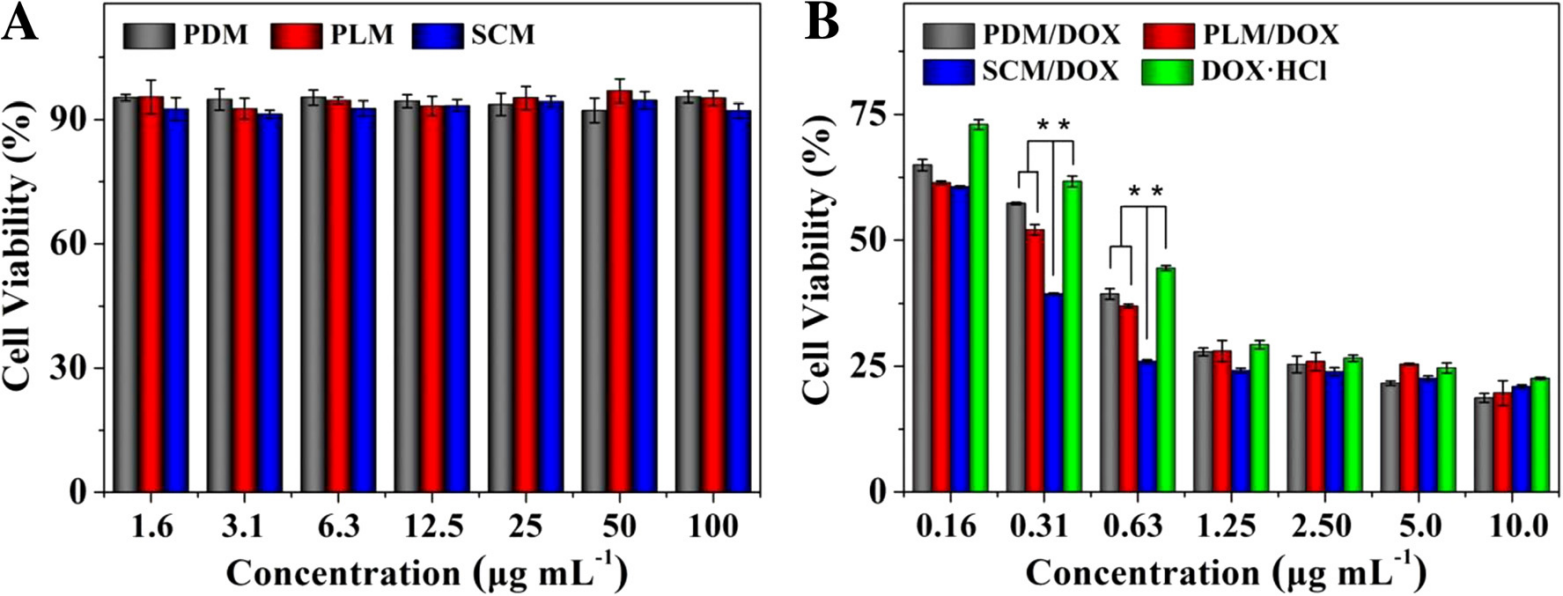
Relative cell viabilities of blank micelles **(A)**, and DOX-loaded micelles and free DOX · HCl **(B)** toward RenCa cells. The data were shown as mean ± SD (*n* = 4; *p < 0.001).

DOX, a widely used small molecule anthracycline antineoplastic agent in clinic, is known to play an important role through the intercalation and inhibition of macromolecular biosynthesis of DNA [29]. The *in vitro* cytotoxicities of blank micelles, DOX-loaded micelles, and free DOX · HCl toward RenCa cells were evaluated by a MTT assay. As depicted in Figure 7A, blank micelles did not show obvious toxicities toward RenCa cells even at a high concentration up to 100.0 μg mL^−1^ after coculturing for 48 h, indicating their excellent biocompatibility. As shown in Figure 7B, compared to free DOX · HCl, the laden micelles appeared a stronger proliferation inhibitory efficacy at equivalent DOX concentration after incubation for 48 h (*p* < 0.001). Moreover, SCM/DOX showed more effective proliferation inhibition efficacy on RenCa cells than those of the DOX-loaded micelles with single component (*p* < 0.001). It might be attributed to a higher extracellular stability of SCM than the micelles with single component. A greater stability guaranteed a larger amount of laden SCM to be internalized into the RenCa cells by endocytosis and more persistent DOX to release in tumor cells, which was consistent with the DOX release profiles of the laden micelles in PBS (Figure 4)*.* At higher DOX · HCl concentrations, that is, 2.5 to 10 μg mL^−1^, it appeared to be little difference in the antineoplastic activity among all the test formulations (*p* > 0.05). It was because that DOX exhibited the highest cytotoxicity with the concentration at around 2.5 μg mL^−1^, and the continuously increased intracellular DOX concentration could not further improve the antineoplastic activities of all the DOX formulations. The half maximal inhibitory concentrations (IC_50s_) of PDM/DOX, PLM/DOX, SCM/DOX, and free DOX · HCl were calculated to be 0.43, 0.34, 0.24, and 0.64 μg mL^−1^, respectively. The lowest IC_50_ also quantitatively proved the more effective antineoplastic efficacy of SCM/DOX.

### Evaluations of serum albumin-tolerance stability and hemocompatibility

**Figure 8:**
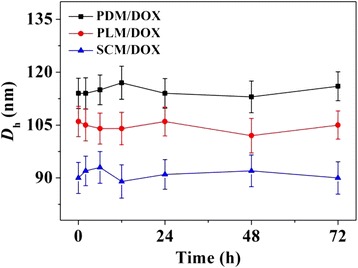
*D*_h_ changes of PDM/DOX, PLM/DOX, and SCM/DOX versus time in PBS-buffered BSA solution (30.0 mg mL^−1^) at pH 7.4, 25°C. Each datum was represented as mean ± SD (*n* = 3).

The evaluations of serum albumin-tolerance stability and hemocompatibility are critical for the DOX-loaded micelles to be applied in clinic because these corresponding formulations are designed to be administrated intravenously [30]. In this study, the stability of these laden micelles was tested by DLS in the PBS-buffered BSA solution at a concentration of 30.0 mg mL^−1^, 25°C. As shown in Figure 8, all the loading micelles exhibited excellent stability among the measurement of 72 h, which were around 115, 105, and 90 nm, respectively. Moreover, the *D*_h_s of these laden micelles in PBS with BSA were similar with the above results in PBS without BSA (Figure 3). The results indicated that the DOX-loaded micelles could keep excellent stability in BSA solution.

**Figure 9:**
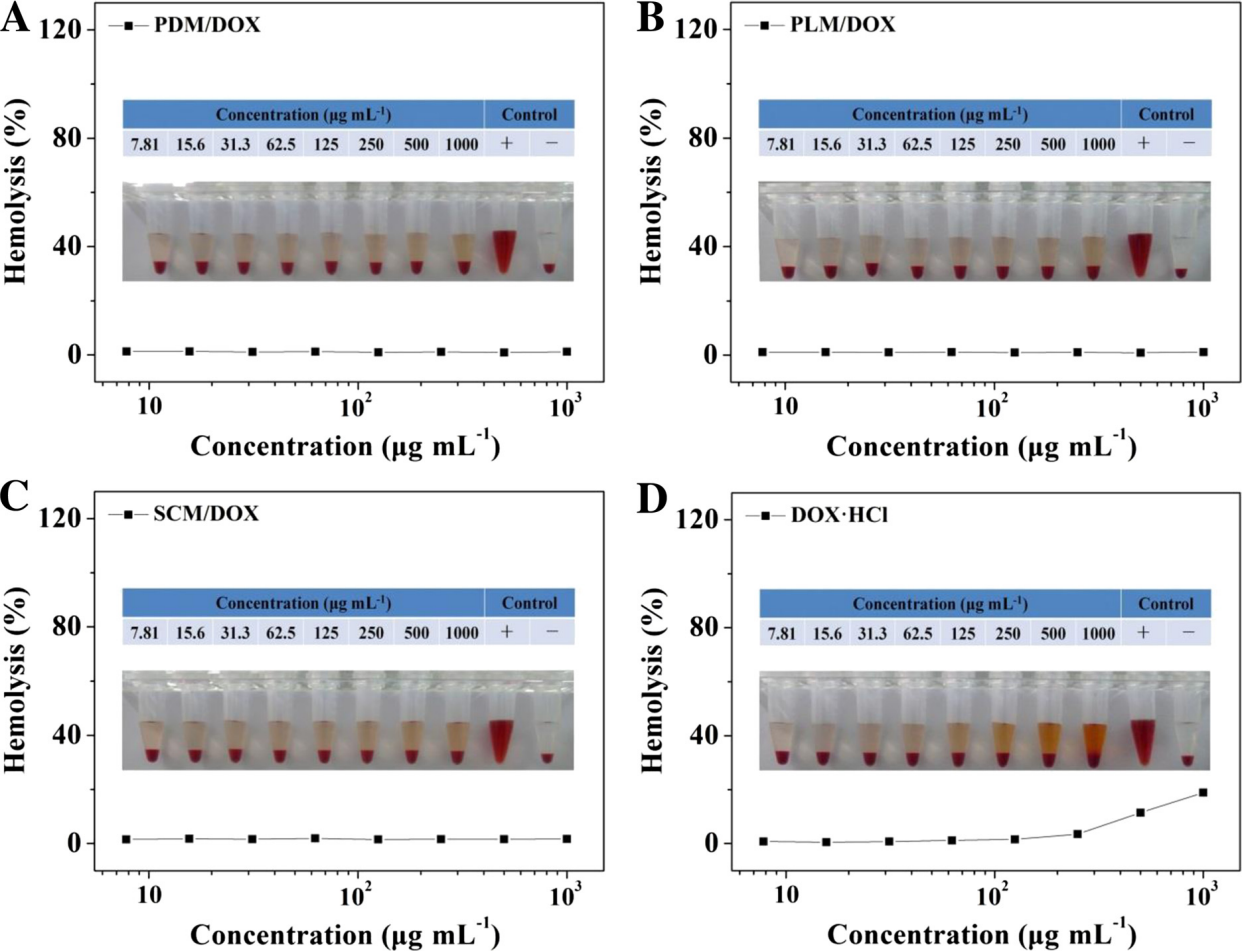
Hemolytic behaviors of PDM/DOX **(A)**, PLM/DOX **(B)**, SCM/DOX **(C)**, and free DOX · HCl **(D)**.

Moreover, as shown in Figure 9, all these laden micelles exhibited no obvious hemolytic activities with the DOX · HCl concentrations up to 1,000 μg mL^−1^. In contrast, free DOX · HCl exhibited more serious hemolytic performance at the concentration over 2.5 mg mL^−1^, which adequately exceeded the final anticipated concentration of drug administrated by intravenous injection *in vivo* [30]. It indicated that the DOX-loaded micelles had satisfactory blood compatibility, which was suitable for the clinical application.

## Conclusions

In this work, the DOX-loaded micelles, i.e., PDM/DOX, PLM/DOX, and SCM/DOX, were successfully designed and prepared from nonlinear enantiomeric PEG-PLA copolymers. The diameters of the loading micelles were approximately 100 nm, which were suitable for the selective accumulation in neoplasm tissue through the EPR effect. Compared to PDM/DOX and PLM/DOX, SCM/DOX exhibited smaller diameter, slower DOX release *in vitro*, and more effective internalization by RenCa cells. Furthermore, SCM/DOX achieved more effective intracellular release of DOX and showed the enhanced cellular proliferation inhibition toward RenCa cells than PDM/DOX, PLM/DOX, and free DOX · HCl. Moreover, all the laden micelles held excellent serum albumin-tolerance stability and exhibited satisfactory hemocompatibility compared to free DOX · HCl. With more effective intracellular DOX release, improved antineoplastic activity, and good biocompatibility, the micelles, especially SCM, might become an effective drug delivery platform of antineoplastic drug for the clinical chemotherapy.
